# From urine to food and oxygen: effects of high and low NH_4_^+^:NO_3_^-^ ratio on lettuce cultivated in a gas-tight hydroponic facility

**DOI:** 10.3389/fpls.2023.1229476

**Published:** 2023-07-31

**Authors:** Mona Schiefloe, Øyvind Mejdell Jakobsen, Antonio Pannico, Claudia Quadri, Ann-Iren Kittang Jost

**Affiliations:** ^1^ Centre for Interdisciplinary Research in Space (CIRiS), NTNU Social Research, Trondheim, Norway; ^2^ Department of Agricultural Sciences, University of Naples Federico II, Portici, Italy; ^3^ EnginSoft Società per Azioni, Bergamo, Italy

**Keywords:** ammonium, nitrate, recycling, oxygen, food production, photosynthesis, life support system

## Abstract

*In situ* production of food, water and oxygen is essential for long-duration human space missions. Higher plants represent a key element in Bioregenerative Life Support Systems (BLSS), where crop cultivation can be based on water and nutrients recovered from waste and wastewater. Human urine exemplifies an important waste stream with potential to provide crops with nitrogen (N) and other nutrients. Dynamic waste composition and treatment processes may result in mineralized fractions with varying ammonium (NH_4_^+^) to nitrate (NO_3_^-^) ratios. In this study, lettuce was cultivated in the unique ESA MELiSSA Plant Characterization Unit, an advanced, gas-tight hydroponic research facility offering controlled environment and continuous monitoring of atmospheric gas composition. To evaluate biological and system effects of nutrient solution NH_4_^+^:NO_3_^-^ ratio, two crop tests were run with different NH_4_^+^ to total N ratio (NH_4_^+^:N) and elevated concentrations of Na^+^ and Cl^-^ in line with a urine recycling scenario. Plants cultivated at 0.5 mol·mol^-1^ NH_4_^+^:N (HiNH_4_^+^) achieved 50% lower shoot biomass compared to those cultivated at 0.1 mol·mol^-1^ NH_4_^+^:N (LoNH_4_^+^), accompanied by higher shoot dry weight content and lower harvest index. Analyses of projected leaf area over time indicated that the reduced biomass observed at harvest could be attributed to a lower specific growth rate during the close-to-exponential growth phase. The HiNH_4_^+^ crop produced 40% less O_2_ over the full cultivation period. However, normalization of the results indicated a marginal increase in O_2_ production per time and per projected leaf area for the HiNH_4_^+^ crop during the exponential growth phase, in line with a higher shoot chlorophyll content. Mineral analysis demonstrated that the biomass content of NH_4_^+^ and NO_3_^-^ varied in line with the nutrient solution composition. The ratio of consumed NH_4_^+^ to consumed N was higher than the NH_4_^+^:N ratio of the nutrient solution for both crop tests, resulting in decreasing NH_4_^+^:N ratios in the nutrient solution over time. The results provide enhanced insight for design of waste processes and crop cultivation to optimize overall BLSS efficiency and hold valuable potential for improved resource utilization also in terrestrial food production systems.

## 1 Introduction

Human spaceflight missions to remote locations call for on-site food production and resource recirculation. In contrast to Low Earth Orbit, complete resupply of resources from Earth is challenged by increased mission duration and travel distances. Higher plants represent a key element for regeneration of air, water and food for astronauts (Wheeler et al., 2002; Pannico et al., 2022), either as part of stand-alone Bioregenerative Life Support Systems (BLSS), or in a combination with physicochemical methods. The MELiSSA project (Micro Ecological Life Support System Alternative) is an international collaboration led by the European Space Agency (ESA), emphasizing the development of a BLSS to support long-term space missions. In an integrated loop, waste streams from a crew compartment are to be processed by thermophilic, heterotrophic and nitrifying bacteria to provide input to photosynthetic compartments with higher plants and algae to regenerate mineralized nutrients and carbon dioxide (CO_2_), and produce food, pure water, and oxygen (O_2_) for the crew (Godia et al., 2002). An important objective of the MELiSSA project is to develop mathematical models that can predict critical processes such as plant growth, photosynthesis, and transpiration, based on parameters such as plant species, cultivation conditions, and the processed waste stream fed to the plants.

In a scenario of crop cultivation based on mineralized human waste, urine is a valuable resource due to its high nitrogen (N) content. N is an essential element and a critical input factor for crop cultivation, with profound effects on plant growth and development as a constituent of numerous vital compounds such as nucleic acids, chlorophyll, amino acids, proteins, ATP, phytohormones, auxin and cytokinins (Marschner and Marschner, 2012; Taiz, 2015; De Bang et al., 2021). The use of recycled organic waste for crop cultivation typically requires mineralization of organic compounds by physicochemical and/or microbial processes. N is primarily taken up by plant roots as inorganic ammonium (NH_4_^+^) and nitrate (NO_3_^-^) (Marschner and Marschner, 2012; Taiz, 2015), that may be derived from urine. While fresh urine is rich in urea-N (up to 12 g L^-1^), this compound is rapidly hydrolyzed upon storage in a non-sterile environment to NH_4_^+^/NH_3_ that may be further converted to NO_3_^-^ by physicochemical and/or microbial processes (Udert et al., 2006; Larsen et al., 2021). As the result of this upstream treatment depends on the mineralization strategy and the process conditions, the resulting nutrient solution provided to the plants may vary in the absolute concentrations of NH_4_^+^ and NO_3_^-^ and the ratio between them. This may in turn affect not only the plant uptake rates of these inorganic N species, but also other physiological and metabolic processes such as uptake of other nutrients, enzyme activity, photosynthesis and respiration, water balance, signaling pathways, leaf expansion, and root architecture - eventually influencing the overall plant growth and crop yield (Guo et al., 2007a; Guo et al., 2007c; Andrews et al., 2013; Liu and Von Wiren, 2017). A nutrient solution containing both NH_4_^+^ and NO_3_^-^ is typically preferred to optimize plant growth and development rather than using either NO_3_^-^ or NH_4_^+^ as the sole source of N, although the use of NH_4_^+^ as the dominant N source should be avoided to reduce risk of ammonium toxicity and reduced plant growth (Roosta and Schjoerring, 2008; Taiz, 2015; Song et al., 2021; Weil et al., 2021; Hameed et al., 2022). Beyond N, urine contains other plant macronutrients such as potassium, phosphorus, sulfur, and lower levels of calcium and magnesium (Udert et al., 2006; Larsen et al., 2021). On the other hand, feeding crops with processed urine introduces non-essential elements such as sodium (Na^+^) and chloride (Cl^-^). Plants naturally accumulate salts, and the presence of Na^+^ and Cl^-^ in the nutrient solution may affect plant physiology and growth. At some concentrations, NaCl has been demonstrated to act as a eustressor with beneficial effects on crop quality (Kronzucker et al., 2013; Rouphael et al., 2018). However, high concentrations of salt is among the most limiting factors for plant growth and may cause injurious abiotic stress, altering morphological and physiological plant traits and ultimately reducing crop yield (Chinnusamy and Zhu, 2006; Acosta-Motos et al., 2017).

To make possible a new generation of scientific studies related to the higher plant compartment, one of the most complex compartments in the MELiSSA loop, a Plant Characterization Unit (PCU) was recently designed and assembled on the premises of Department of Agricultural Sciences of University of Naples Federico II in Italy as part of the ESA MELiSSA PaCMan (PlAnt Characterization unit for closed life support system – engineering, MANufacturing & testing) activity (Pannico et al., 2022). The PCU is a gas-tight cultivation chamber for characterization of higher plants, offering extensive monitoring and control of the cultivation environment, including its separated hydroponic and atmospheric loops.

Lettuce is a highly relevant species for bioregenerative life support systems with high harvest index, efficiency (per area, time, and volume), and potential yield (edible biomass, O_2_ and water), combined with relatively modest horticultural requirements (Berkovich et al., 2004). Lettuce has been successfully cultivated onboard the International Space Station (Khodadad et al., 2020) and represents a healthy and nutritious addition to the human diet, rich in vitamin C, antioxidants, polyphenols and dietary fiber (Khodadad et al., 2020). Furthermore, it is one of the most used vegetables in terrestrial hydroponic cultivation systems (Zhu et al., 2020), illustrating the benefits of a deeper understanding of plant responses to nutrient solutions derived from organic waste in commercial crop production also on Earth, with the potential to improve resource utilization and reduce environmental impact of industrial food production systems.

The underlying hypotheses and scenario of this study was that strategies and operating conditions of upstream waste processing will affect the composition of the mineralized nutrients fed to the plants in a life support system. As these strategies and conditions may be designed and controlled, but also give rise to fluctuations and dynamics, it is critical to understand and predict consequences of nutrient solution composition on crop cultivation. More specifically, urine ammonification and nitrification may result in nutrient solutions with varying concentrations of NH_4_^+^ and NO_3_^-^, including their relative ratio, which will impact downstream crop cultivation. Furthermore, beyond the incoming nutrient streams, nutrient utilization rates inside the plant compartment will dictate the evolution of the nutrient solution in a closed-loop scenario with complete recycling of water, nutrients and non-nutrients. This study aimed at investigating biological and system effects of high and low NH_4_^+^:N ratios on lettuce cultivated in a urine recycling scenario, exploiting the recently established PCU facility offering controlled hydroponic cultivation conditions and state of the art monitoring of critical parameters such as O_2_ production.

## 2 Materials and methods

### 2.1 Lettuce seedling production

About 100 lettuce seeds (*Lactuca sativa* L. cultivar ‘Grand Rapids’) were disinfected in 5% sodium hypochlorite solution for 15 minutes followed by three rinsing cycles in ultrapure water. The disinfected seeds were dispersed onto absorbent paper, moist with ultrapure water and incubated at room temperature under indirect lighting. After 48 hours, germinated seeds with about 1 cm radicle were transferred to Styrofoam trays with vermiculite soaked in nutrient solution prepared according to Peiro et al. (2020) at 0.5X strength, and incubated at 24°C in a dedicated nursery with 65% relative humidity (RH), a light/dark regime of 16/8 hours and a light intensity of 400 ± 50 μmol·m^–2^·s^–1^ (PPFD). Ten days after sowing, 18 equally developed seedlings (evaluated by visual inspection) were selected from the pool of 100 germinated seeds. Each seedling was installed into a bottomless 50 mL test tube (CLS430829, Corning, NY, USA) filled with rockwool (Delta 4G 42/40, Grodan, Roermond, The Netherlands) cut into a cylindrical shape and vertically split in two. The seedling’s root system was sandwiched between the two halves of the rockwool column and inserted into the 50 mL tube. The top surface of the rockwool was then covered with grafting mastic (Cortimax, Cisa Adriatica S.r.l., Pescara, Italy) to minimize gas exchange between the root and shoot zones of the cultivation chamber.

### 2.2 Cultivation chamber and cultivation conditions

For each of the two crop tests (designated HiNH_4_^+^ and LoNH_4_^+^), 18 lettuce plants were cultivated inside the PCU. Within one crop test, all plants shared the same nutrient solution (recirculated within the PCU’s hydroponic loop) and the same atmosphere (recirculated within the PCU’s atmospheric loop). The plants were cultivated for 28 days inside the PCU chamber to a total plant age of 38 days. The day/night cycle was 16/8 hours, with an average light intensity across the plant positions of 430 µmol·m^-2^·s^-1^ (PPFD represented by 23% blue, 20% green and 57% red light). PPFD and spectral composition were recorded by eighteen spectral scans (one per plant position, measured at a height of 3 cm above the medium surface) using a spectral radiometer (MSC15, Gigahertz-Optik, Türkenfeld, Germany). The largest deviation between this average light intensity and the intensity at any of the plant positions was 68 µmol·m^-2^·s^-1^ (PPFD), corresponding to 16% of the average intensity. The air temperature and RH were automatically controlled as described by Pannico et al. (2022), at 26°C and 50% during the day and 20°C and 70% during the night, respectively. The atmospheric CO_2_ concentration was controlled at 1000 ppmv (parts per million by volume) by automatic injection of pure CO_2_ during the day phase, while it was allowed to increase during the night phase due to respiration. Atmospheric O_2_ concentration started at ambient levels and increased based on plant photosynthesis. The atmospheric O_2_ concentration was not controlled beyond one chamber venting per crop test (Day 19) to avoid O_2_ levels rising above 25%.

### 2.3 Nutrient solution preparation, monitoring, and maintenance

The nutrient solutions were based on the recipe described by Peiro et al. (2020) with the exception of i) the NH_4_^+^:N ratio was either reduced (LoNH_4_^+^ crop test) or increased (HiNH_4_^+^ crop test) using SO_4_^2-^ as counter-ion for required charge balancing, and ii) Na^+^ and Cl^-^ levels were increased (both crop tests). Identification of prioritized nutrient solutions for the two PCU crop tests was based on preliminary deep water crop cultivation experiments with various NH_4_^+^:N ratios (0.00, 0.11, 0.22, 0.44, 0.50 mol·mol^-1^), nutrient solution strengths (0.75, 1.00, 1.25 X) and NaCl concentrations (0, 5 mM). PCU crop cultivation conditions were selected aiming at the presence of NaCl (5 mM) to mimic a urine recycling scenario (see Results section), reduced nutrient solution strength (0.75 X) to minimize negative effects of elevated total ionic strength (maintaining same electrical conductivity for the LoNH_4_^+^ crop test as for the nutrient solution of Peiro et al. (2020), 1.9 mS/cm), and two NH_4_^+^:N ratios representing considerable differences (high and low) while still providing acceptable plant growth conditions and representing relevant degrees of nitrification in a BLSS perspective (see Results section). The nutrient solutions of the LoNH_4_^+^ and HiNH_4_^+^ crop tests were obtained by mixing the following A and B stock solutions made from reverse osmosis water: LoNH_4_^+^ Stock A consisted of 434 mM Ca(NO_3_)_2_, 90 mM CaCl_2_, 9.6 mM FeCl_3_ and 12 mM Na-EDTA. HiNH_4_^+^ Stock A consisted of 213 mM Ca(NO_3_)_2_, 311 mM CaCl_2_, 9.6 mM FeCl_3_ and 12 mM Na-EDTA. LoNH_4_^+^ Stock B consisted of 120 mM MgSO_4_, 420 mM KNO_3_, 180 mM KH_2_PO_4_, 207 mM NH_4_NO_3_, 187 mM NaNO_3_, 590 mM NaCl, and micronutrients. HiNH_4_^+^ Stock B consisted of 120 mM MgSO_4_, 420 mM KNO_3_, 415 mM (NH_4_)_2_SO_4_, 180 mM KH_2_PO_4_, 107 mM NH_4_NO_3_, 315 mM Na_2_SO_4_, 145 mM NaCl, and micronutrients. Micronutrients (part of Stock B) were 2.4 mM H_3_BO_3_, 0.42 mM ZnSO_4_, 96 µM CuSO_3_, 0.6 mM MnSO_4_, and 60 µM H_2_MoO_4_. For the LoNH_4_^+^ crop test, LoNH_4_^+^ A and B stock solutions were added at a 1:1 ratio to achieve an electrical conductivity of 1.9 mS·cm^-1^ at startup, similar to that of Peiro et al. (2020). This resulted in a total N concentration of 11 mM. For the HiNH_4_^+^ crop test, HiNH_4_^+^ A and B stock solutions were added at a 1:1 ratio to achieve the same total N concentration (11 mM). This resulted in an electrical conductivity of 2.2 mS·cm^-1^ at startup, due to higher concentrations of SO_4_^2-^ used for charge balancing. The measured ion concentrations, pH and EC of both nutrient solutions are summarized in **Table 1**. The total liquid volume in the hydroponic loop was 265 - 275 L throughout both crop tests.

**Table 1:** Characterization of the LoNH_4_^+^ and HiNH_4_^+^ nutrient solutions at start (0 days after transplant (DAT)) and end (28 DAT) of each crop test.

	LoNH_4_^+^		HiNH_4_^+^	
	0 DAT	28 DAT	0 DAT	28 DAT
NO_3_^-^ [mM]	9.7	9.7	5.6	5.6
NH_4_^+^ [mM]	1.3	0.2	5.3	1.6
NO_2_^-^ [mM]	0.0	0.0	0.0	0.5
Total N [mM]	11.0	10.0	10.9	7.8
HPO_4_^2-^ [mM]	0.8	0.7	0.9	0.7
K^+^ [mM]	3.7	3.1	3.5	7.4
Ca^2+^ [mM]	4.6	6.5	4.8	4.6
Mg^2+^ [mM]	0.7	0.8	0.7	0.6
SO_4_^2-^ [mM]	0.8	0.9	4.9	4.7
Cl^-^ [mM]	4.5	5.2	4.7	4.2
Na^+^ [mM]	5.1	6.4	4.9	4.8
NH_4_^+^:NO_3_^-^ [mol·mol^-1^]	0.14	0.03	0.95	0.28
NH_4_^+^:N [mol·mol^-1^]	0.12	0.02	0.49	0.20
Na:N [mol·mol^-1^]	0.46	0.64	0.46	0.62
Cl:N [mol·mol^-1^]	0.41	0.52	0.43	0.54
K:N [mol·mol^-1^]	0.33	0.31	0.32	0.96
pH	5.9	5.9	5.9	5.9
EC [mS·cm^-1^]	1.9	1.9	2.2	2.3

Water quality of the recirculating nutrient solution was continuously monitored, including pH (2 x Endress+Hauser Orbisint CPS11D Memosens, Germany), electrical conductivity (EC; 2 x Endress+Hauser Indumax CLS50D, Germany), dissolved O_2_ (Mettler Toledo InPro 6860i, Switzerland), dissolved CO_2_ (Labolytic optical CO_2_ sensor 0-15 mg/L, Trondheim, Norway), and nitrate (Endress+Hauser Viomax CAS51D, Germany, installed in an at-line configuration with auto-sampling and auto-dilution of the nutrient solution). Nutrient solution temperature was continuously monitored and automatically controlled at 18 ± 0.5°C. For both crop tests, pH was adjusted to 5.9 at startup using 0.5 M H_2_SO_4_ and then maintained at 5.9 by automatic addition of 0.5 M KOH throughout both crop tests (no addition of acid necessary during the cultivation period). Throughout both crop tests, the actual pH of the nutrient solution typically deviated less than 0.05 pH units from the set-point, and never more than 0.2 pH units. During both crop tests, nutrient stock solutions A and B were automatically fed at a 1:1 ratio to maintain a constant NO_3_^-^ concentration. The recirculating nutrient solution was mechanically filtered in three steps with reducing pore size from 1.6 to 0.45 µm, using filter cartridges (PP3 Sartopure - Sartobran, Sartorius, Goettingen, Germany). The filtration unit was implemented between the plant cultivation chamber and the stirred tank holding the nutrient solution that was continuously fed to the plants.

### 2.4 Data collection, sampling, and analysis

Atmospheric concentrations of O_2_ and CO_2_ inside the cultivation chamber were continuously measured by an O_2_/CO_2_ gas analyzer (California Analytical Instruments, 700 LX NDIR/O_2_, Orange, CA, USA). Additionally, the O_2_ and CO_2_ concentrations of the pressure compensation system’s gas tank were measured at crop test start and end, and before and after chamber venting. The amount of atmospheric gas inside the PCU was determined continuously based on ideal gas law, a total atmospheric volume of 4.9 m^3^, and continuously measured gas pressure and temperature. This allowed computation of daily atmospheric leak rates and plant O_2_ production (Pannico et al., 2022). To keep the air humidity constant, the water produced by the plant transpiration was condensed and collected (Pannico et al., 2022). During the crop tests presented in this study, the injection rates of CO_2_ and water into the atmospheric loop were inaccurate, unfortunately preventing accurate mass balances for CO_2_ and water and thereby also preventing calculation of consumed amounts of CO_2_ and produced amounts of water.

Top-view images of the 1.2 m × 1.5 m cultivation chamber floor were acquired every hour (FLIR Blackfly S BFS-U3-200S6C-C @ 70 dpi), and projected leaf area as observed from above was calculated for images acquired every second day based on image segmentation by color. Projected Leaf Area Index (PLAI) was calculated as the measured projected leaf area of all 18 plants (m^2^) per total cultivation area (m^2^) (Zheng and Moskal, 2009). SPAD index for indication of chlorophyll content (average of ten measurements per plant; SPAD-502, Minolta Corp. Ltd, Osaka, Japan), dark-adapted chlorophyll fluorescence for the maximum quantum yield of photosystem II (F_v_/F_m_) (average of two measurements per plant; Plant Stress Kit, Opti-Sciences), and leaf color index (average of three measurements per plant; Chroma Meter CR-400, Konika Minolta) were measured immediately after opening the cultivation chamber at day of harvest. Fresh weight of shoot and stem, and leaf number were determined for each plant by destructive sampling. Additionally, total leaf area of each plant was determined from top-view images of the plant’s individual leaves (Canon EOS 90D + Tamron SP 35 mm @ 59 dpi) based on image segmentation by color.

The lead taproot of each plant was isolated and imaged (Canon EOS 90D + Tamron SP 35 mm @ 294 dpi for LoNH_4_^+^ and 334 dpi for HiNH_4_^+^) in a tray with water. The lead taproot was defined as the medial root with the largest diameter in the basal zone, not considering the diameter of the laterals. Root length, diameter and number of links and forks were analyzed with WinRhizo (Pro 2021a, Regent Instruments Inc.). Following counting of leaf number and determination of shoot fresh weight (FW) of each plant, shoots and roots were oven-dried at 60°C until constant weight was reached for determination of dry weight (DW) per plant. Total plant DW was calculated as shoot DW + root DW, while harvest index was calculated as shoot DW per total plant DW. Dried samples of shoots and roots were analyzed for total carbon (C) and N content via combustion analysis by a Micro Elemental Analyser UNICUBE^®^ (Elementar, Langenselbold Hesse, Germany) equipped with a thermal conductivity detector. Leaf soluble cations and anions were determined by liquid ion exchange chromatography (ICS-3000, Dionex, Sunnyvale, CA, USA) as described by Pannico et al. (2019).

Every two days, nutrient solution samples (25 mL) were extracted from the hydroponic loop for offline analysis. Nutrient ion concentrations were determined by ion exchange chromatography (ICS 3000 Dionex) and the results were smoothed by moving average to filter out noise from analysis uncertainty. N mass balance was calculated based on measured parameters at the start and end of each crop test, including concentrations of inorganic N in the nutrient solution (measured via optical sensor and ion chromatography methods described above), total liquid volume, total shoot and root biomass and their N content, in addition to the amount of nutrient stock solutions introduced and samples removed throughout the crop test.

Statistical analyses were performed with SPSS (IBM SPSS version 28.0.0.0), Anova with Tukey HSD post-test, tests of normality, Independent Samples t-test and Mann Whitney U test.

## 3 Results

Building on the background presented in the introduction, the results section first presents the experiment rationale with respect to nutrient solution composition and cultivation conditions. Representing the first scientific paper detailing crop cultivation results from the recently established PCU facility, plant growth and selected scientific datasets offered by this facility are then illustrated on a high level, before being explored in more detail to present effects of crop cultivation in low vs. high NH_4_^+^:N ratio.

### 3.1 Urine recycling scenario: Experiment rationale and nutrient solution design

The experiment rationale was built on the hypothesis presented in the introduction that both design and natural dynamics of upstream waste processing may result in mineralized waste streams with different NH_4_^+^:NO_3_^-^ ratios, which in turn may affect the biology of a downstream plant compartment and thus the overall BLSS performance. Effects of different NH_4_^+^:NO_3_^-^ ratios in the nutrient solution were explored in a mineralized urine scenario, using NH_4_^+^ and NO_3_^-^ as the sole N sources. Two crop tests (two treatments), designated LoNH_4_^+^ and HiNH_4_^+^, were performed with NH_4_^+^ to total N ratios (NH_4_^+^:N) of 0.1 and 0.5 mol·mol^-1^, respectively. Relating to a foreseen upstream nitrification process, the LoNH_4_^+^ scenario (0.1 mol·mol^-1^ NH_4_^+^:N) represents nearly complete nitrification, while the HiNH_4_^+^ scenario (0.5 mol·mol^-1^ NH_4_^+^:N) represents nitrification without increased alkalinity and thus limited by redox, as reviewed by Larsen et al. (2021). At the same time, these scenarios represent considerable different, low and high NH_4_^+^:N ratios for lettuce cultivation (Zhu et al., 2020; Du et al., 2022; Hameed et al., 2022) while still avoiding the use of NH_4_^+^ as the dominant N source to limit the potential of ammonium toxicity and thereby allow acceptable growth (see Materials and methods). To allow direct comparison of results, total N concentration was identical at startup of both crop tests (11 mM). Capitalizing on a state-of-the-art nutrient monitoring and control system, the NO_3_^-^ concentration was kept constant (9.7 mM in LoNH_4_^+^; 5.6 mM in HiNH_4_^+^) throughout each of the two crop tests to minimize effects of the absolute NO_3_^-^ concentration itself. This allowed the NH_4_^+^:N ratio to change throughout the cultivation period depending on the total consumption of NH_4_^+^ and NO_3_^-^ in the system, demonstrating the development of the NH_4_^+^:N ratio over time.

In line with a urine recycling scenario, the crop tests were performed with an elevated NaCl concentration of 5 mM, representing a Na:N concentration ratio of 0.46 mol·mol^-1^. This ratio was designed based on typical Na:N ratios of mineralized urine. While fresh urine is typically characterized by 0.17 mol·mol^-1^ Na:N and Cl:N (adapted from Udert et al., 2006), several reports illustrate ratios of approximately 0.25 mol·mol^-1^ in urine-based fertilizer solutions (adapted from Bonvin et al., 2015; Mauerer et al., 2018; Halbert-Howard et al., 2021). However, the final composition of the mineralized nutrient solutions depends on urine storage and ammonification and nitrification processes. As urine is prone to ammonia volatilization, resulting Na:N and Cl:N ratios may be considerably elevated - exemplified by the real stored urine fraction by Udert and Wachter (2012) with Na:N and Cl:N ratios of 0.5 mol·mol^-1^. Thus, the Na:N and Cl:N concentration ratios used in this study represent a urine-based nutrient solution, accounting for effects by urine storage and processing. However, effects of potential Na^+^ accumulation over time in a closed loop system are not accounted for in this study, as such predictions would benefit from more insight in Na uptake ratios as function of nutrient solution composition and cultivation conditions.

Beyond NH_4_^+^/NO_3_^-^ and Na^+^/Cl^-^, the two crop tests were run with similar concentrations of macro- and micronutrients, except for SO_4_^2-^ used for charge balancing of NH_4_^+^/NO_3_^-^. The total strength of the nutrient solutions was designed so that the total electrical conductivity of the LoNH_4_^+^ nutrient solution at start was identical to that of Peiro et al. (2020) (1.9 mS·cm^-1^) used for similar MELiSSA studies of hydroponic lettuce cultivation (resulting in an electrical conductivity of 2.2 mS·cm^-1^ for the HiNH_4_^+^ nutrient solution due to the elevated requirement of SO_4_^2-^ for charge balancing). The resulting nutrient solutions based on the above design criteria are illustrated in **Table 1**.

### 3.2 Crop cultivation in the ESA MELiSSA Plant Characterization Unit

**Figure 1:**
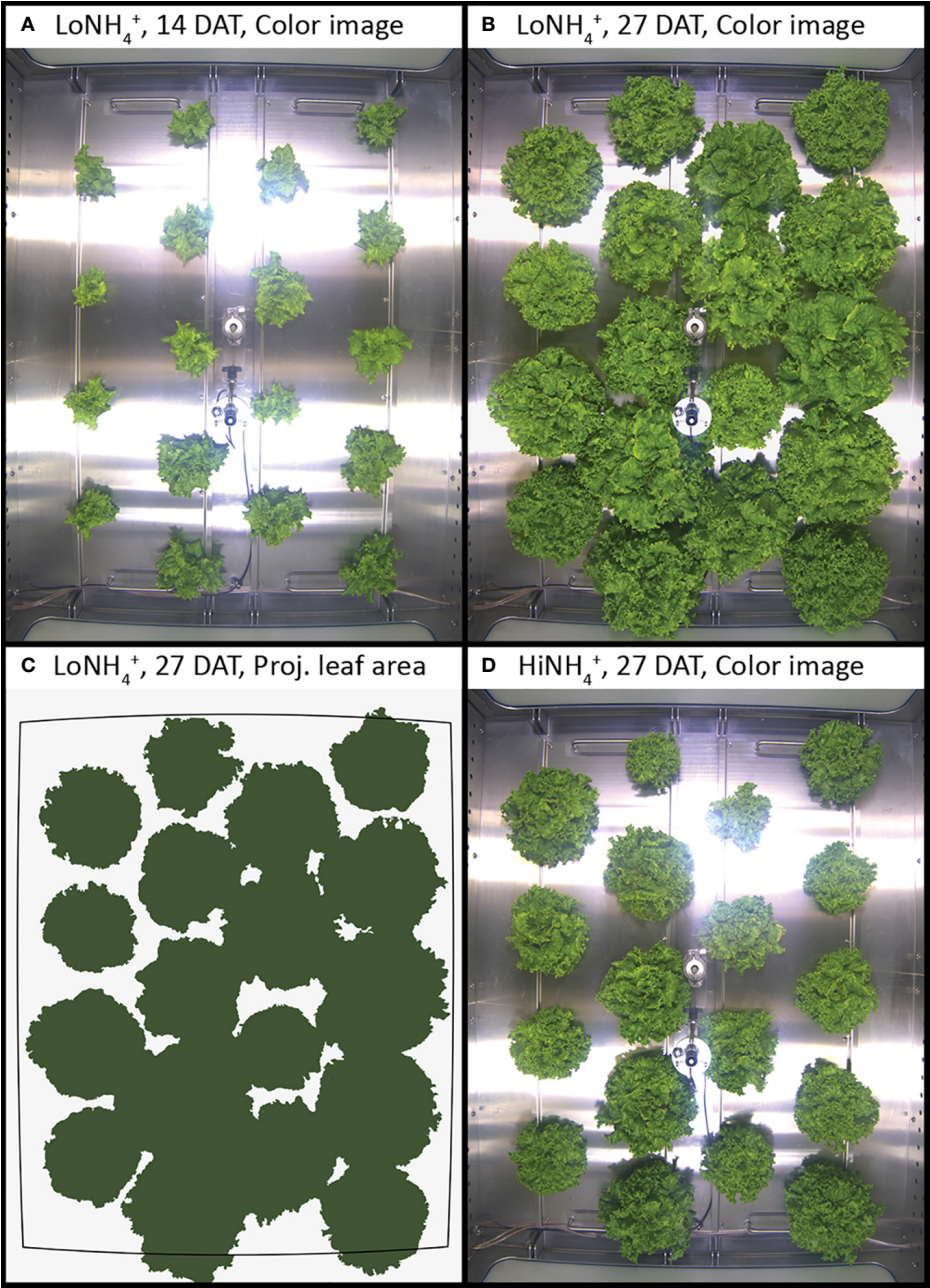
Top-view color images of lettuce inside the PCU cultivation chamber at 14 and 27 days after transplant (DAT), cultivated in LoNH_4_^+^ **(A, B)** and HiNH_4_^+^ nutrient solution **(D)**. Example illustrating image segmentation for determination of projected leaf area **(C)**.

**Figure 2:**
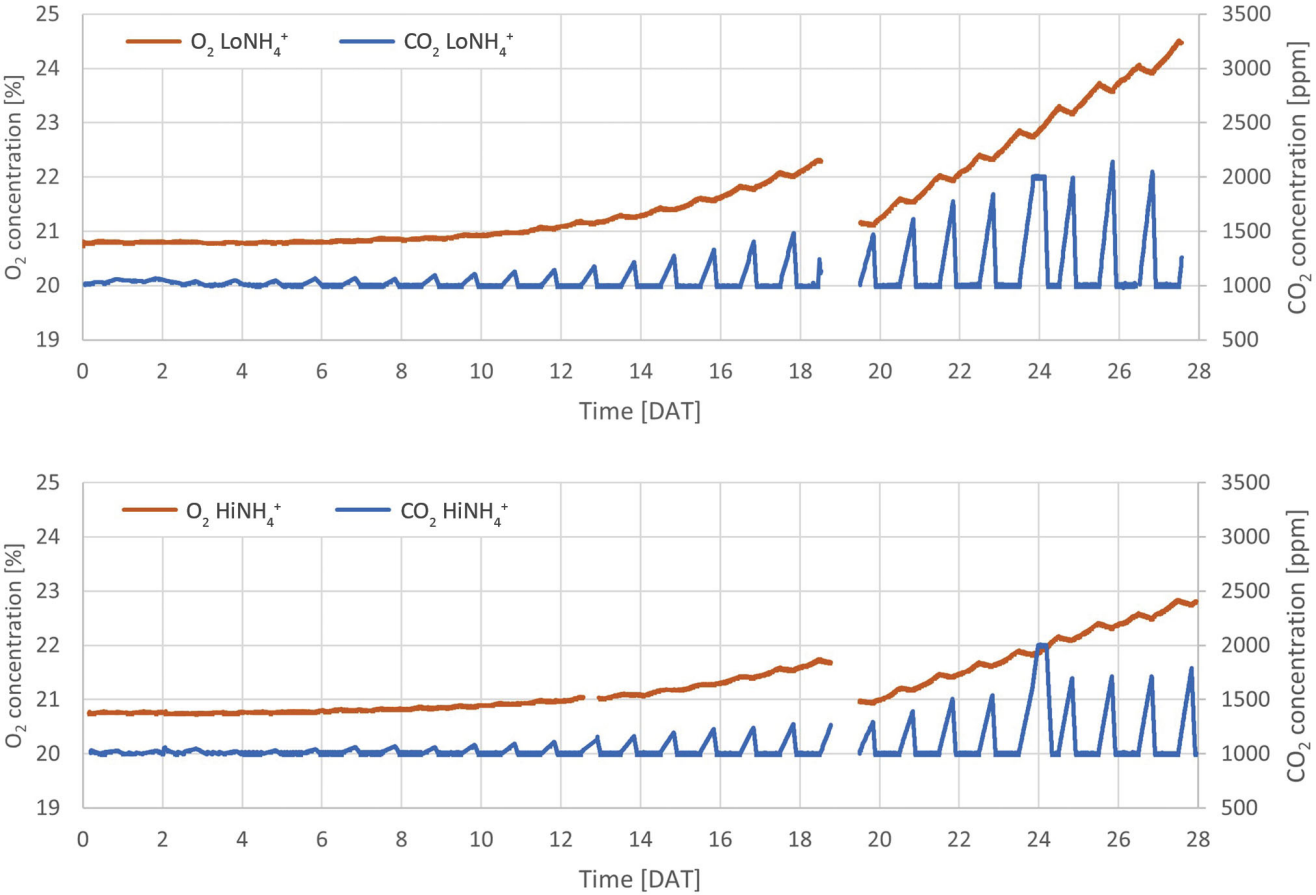
Absolute concentrations of atmospheric O_2_ and CO_2_ throughout the LoNH_4_^+^ (upper graph) and HiNH_4_^+^ (lower graph) crop tests. Missing data from 19 DAT represents a venting event in which the cultivation chamber was opened to reduce the O_2_ concentration.

**Figure 3:**
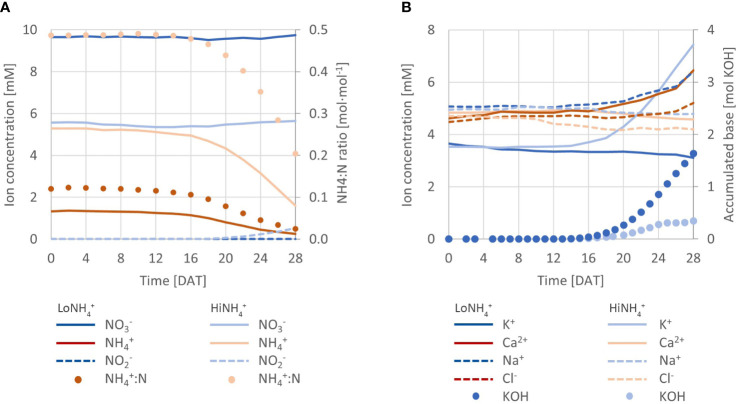
**(A)** Measured concentration of NO_3_^-^, NH_4_^+^, and NO_2_^-^ throughout the LoNH_4_^+^ and HiNH_4_^+^ crop tests (lines; left Y-axis), in addition to the ratio of NH_4_^+^ to total N (NH_4_^+^:N; dots; right Y-axis). **(B)** Development of K^+^, Ca^2+^, Na^+^ and Cl^-^ concentrations in the nutrient solutions of both crop test (lines; left Y-axis), in addition to accumulated addition of base (KOH; dots; right Y-axis).

**Figure 4:**
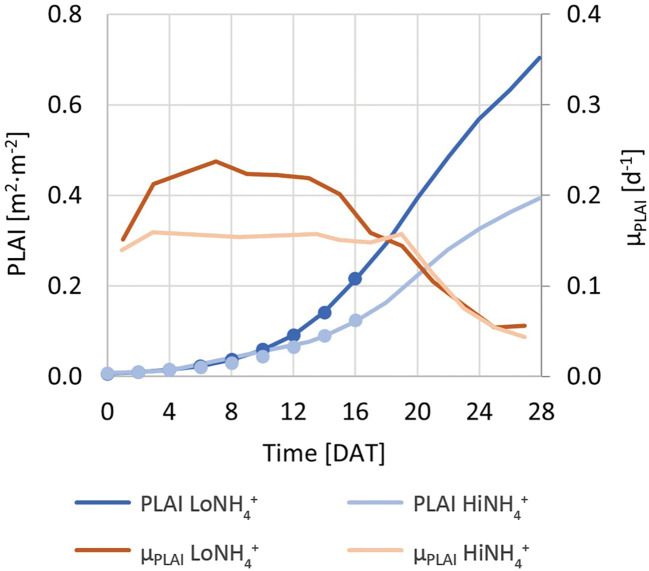
Blue solid lines (left Y-axis): PLAI (Projected Leaf Area Index) of the LoNH_4_^+^ and HiNH_4_^+^ crop tests. Blue circles (left Y-axis): Trendlines based on exponential regression of a subset of the PLAI dataset from 0 to 16 DAT (coinciding with the PLAI dataset in blue solid lines). Red lines (right Y-axis): PLAI-based specific growth rate, µ_PLAI_, of the LoNH_4_^+^ and HiNH_4_^+^ plants.


The results reported in this paper were obtained in the recently established ESA MELiSSA Plant Characterization Unit (PCU), in which 18 lettuce plants were hydroponically cultivated as deep-water culture for 28 days after transplant (DAT), to a total age of 38 days, across a 1.8 m^2^ plant cultivation area (**Figure 1**). Extensive monitoring and control systems provided stable cultivation conditions, including atmospheric temperature and RH, and nutrient solution temperature, pH and NO_3_^-^ concentration (see Materials and methods, **Table 1**; **Figure 3**). The closed nature of the atmospheric loop combined with continuous monitoring of atmospheric O_2_ and CO_2_ concentrations represent the basis for determination of O_2_ production and CO_2_ consumption by the plants. Atmospheric leak rates in percent gas volume per hour were calculated daily and demonstrated a stable and high system tightness with an average leak rate of 0.082 ± 0.017%·h^-1^ and 0.095 ± 0.022%·h^-1^ for the LoNH_4_^+^ and HiNH_4_^+^ crop tests, respectively. The atmospheric O_2_ concentration increased during the day phase resulting from photosynthesis, while it decreased during the night phase due to respiration (**Figure 2**). As the O_2_ production during the day phase exceeds the O_2_ consumption during the night phase, atmospheric O_2_ gradually increased throughout the crop tests creating a need to vent the cultivation chamber to stay below 25% O_2_ for safety reasons (performed at Day 19 for both crop tests). Atmospheric CO_2_ was controlled at a minimum of 1000 ppm during the day phase, while it increased due to respiration during the night phase (up to 2140 ppm during the LoNH_4_^+^ crop test). As the PCU chamber is sealed and only opened for venting and reduction of atmospheric O_2_ concentration, sampling or physical inspection of the plants is not possible during a crop test. To demonstrate biomass development, Projected Leaf Area Index (PLAI) was calculated based on top-view images acquired by the PCU’s internal color camera (**Figure 4**). To further indicate differences in biomass development over time (**Figure 4**) despite the lack of true biomass measurements, PLAI was used as a basis for calculating a specific growth rate, here introduced as µ_PLAI_, according to Equation 1 where t symbolizes time (in DAT).

Equation 1 $${µ}_{PLAI}=\;\frac{\frac{d\left(PLAI\right)}{d\left(t\right)}}{\;\;PLAI\;\;}$$

### 3.3 Plant development and physiology at high and low NH_4_^+^:NO_3_^-^ ratios

#### 3.3.1 Shoot development over time as observed by non-destructive methods

A relatively stable µ_PLAI_ during the first 16 - 18 days indicates close to exponential growth for plants in both the HiNH_4_^+^ crop test and the LoNH_4_^+^ crop test in this period. From approximately 20 DAT, as the plants mature and start to overlap, the PLAI-based specific growth rates of both treatments coincide and decrease (**Figure 4**). Regression analysis of PLAI from 0 to 16 DAT was performed using exponential trendlines (**Figure 4**; R^2^ factors > 0.999), demonstrating a reduced µ_PLAI_ for plants growing in the HiNH_4_^+^ nutrient solution (0.18 per day) compared to that of LoNH_4_^+^ (0.22 per day). The initial PLAI of HiNH_4_^+^ and LoNH_4_^+^ plants were similar due to identical germination and seedling phases (both 0.010 m^2^·m^-2^ at 2 DAT). The final PLAI of the LoNH_4_^+^ crop test at 28 DAT reached 0.70 m^2^·m^-2^ corresponding to a projected leaf area of 1.3 m^2^, while corresponding values of HiNH_4_^+^ were 44% lower with 0.39 m^2^·m^-2^ PLAI and 0.71 m^2^ projected leaf area. Closer inspection of the PLAI of the two crops illustrate that their differences stayed below approximately 20% during the first 10 days in the PCU, before it increased gradually up to approximately 45% at 18 DAT and remained at this level throughout the rest of the cultivation period.

#### 3.3.2 Root and shoot biomass and morphology at harvest

In line with the differences demonstrated by non-destructive PLAI analysis, detailed inspection of the plants after destructive sampling at 28 DAT illustrated a total leaf area of the HiNH_4_^+^ crop which was 50% lower than that of the LoNH_4_^+^ crop (3.5 m^2^ vs. 7.0 m^2^, respectively, for the 18 plants; **Table 2**). The results demonstrate that the shoot biomass was affected in a similar way, with the HiNH_4_^+^ plants having 58% lower shoot fresh weight and 40% lower shoot dry weight compared to the LoNH_4_^+^ plants. The difference in these numbers illustrate a higher shoot dry weight content of the HiNH_4_^+^ plants compared to the LoNH_4_^+^ plants (10% vs. 7%, respectively). Furthermore, the HiNH_4_^+^ plants developed a significantly lower number of leaves (35%) compared to the LoNH_4_^+^ group.

**Table 2:** Biomass characteristics of plants from the LoNH_4_^+^ and HiNH_4_^+^ crop tests, measured at harvest (28 DAT).

	LoNH_4_^+^	HiNH_4_^+^	Statistical significance
Shoot fresh weight per plant [g]	289 ± 122	121 ± 51	p < 0.01
Shoot dry weight per plant [g]	19 ± 5	11 ± 4	p < 0.01
Shoot dry weight content [%]	6.9 ± 1.3	9.8 ± 1.6	p < 0.01
Root dry weight per plant [g]	2.4 ± 0.7	1.9 ± 0.6	p < 0.05
Total dry weight per plant [g]	21 ± 6	13 ± 4	p < 0.01
Harvest index	0.88 ± 0.02	0.85 ± 0.02	p < 0.01
Root:shoot ratio	0.14 ± 0.02	0.19 ± 0.03	p < 0.01
Stem diameter per plant [mm]	18 ± 3	15 ± 3	p < 0.01
Leaf number per plant	40 ± 19	26 ± 8	p < 0.05
Total leaf area per plant [cm^2^]	3907 ± 724	1961 ± 712	p < 0.01

All values are tabulated as mean value ± standard deviation based on separate analyses of 18 individual plants from each crop test. Statistical significance between the two crop tests is indicated as p-values. The shoot dry weight content was calculated as shoot dry weight divided by shoot fresh weight, the harvest index was calculated as shoot dry weight divided by total dry weight, while the root:shoot ratio was calculated based on dry weight.

**Figure 5:**
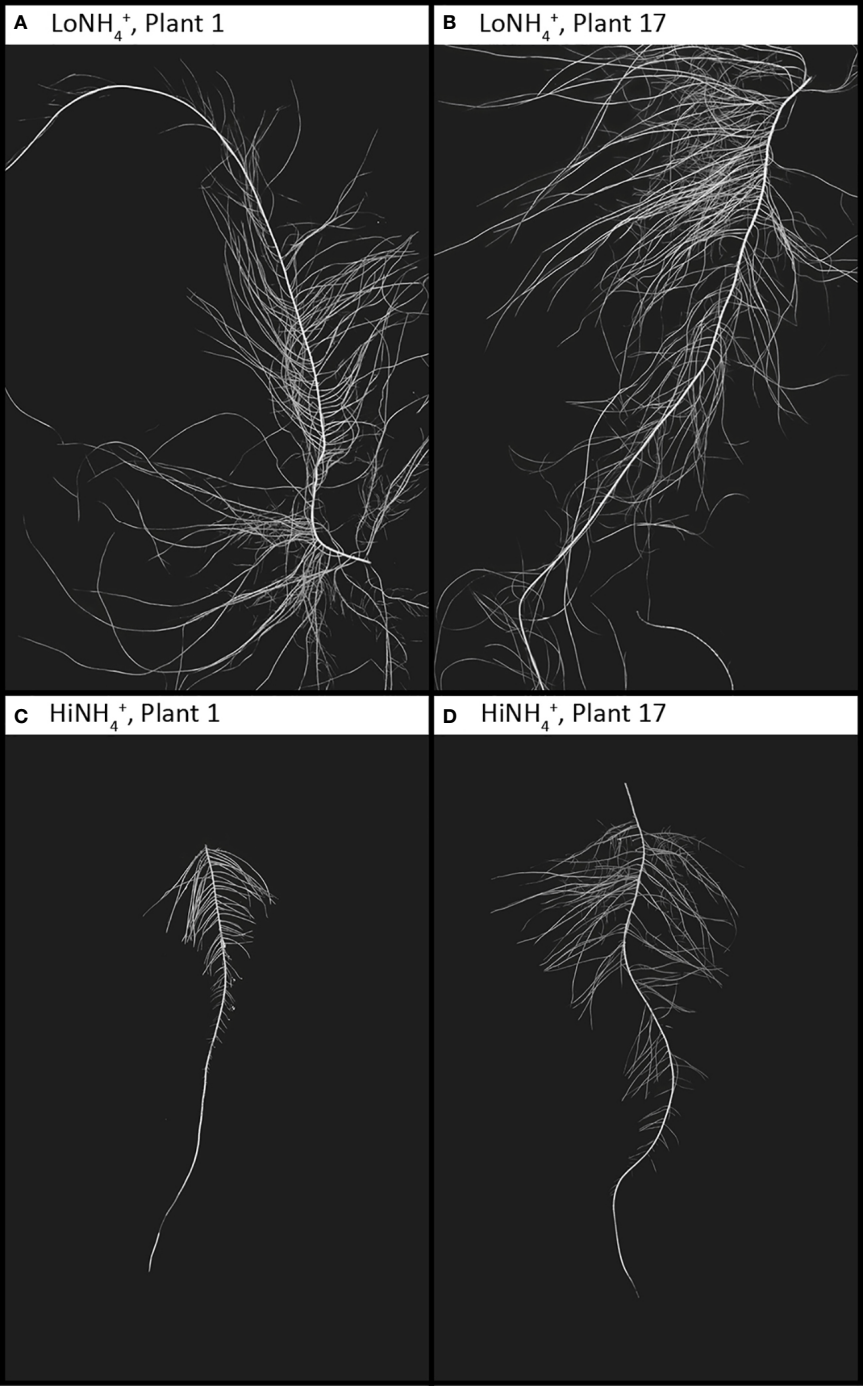
Lead taproots of two example LoNH_4_^+^ plants **(A, B)** and two HiNH_4_^+^ plants **(C, D)** from harvest at 28 DAT. All images are represented at the same resolution, covering a 20 x 30 cm area. Root and background contrast and color levels have been modified from the raw images to enhance root morphology.

The dry root biomass difference between the two crops (19% lower for HiNH_4_^+^ plants) was smaller than the shoot dry biomass difference (40% lower for HiNH_4_^+^), illustrating a higher root:shoot ratio and a lower harvest index of the HiNH_4_^+^ plants compared to those of LoNH_4_^+^ (**Table 2**). Following harvest and destructive sampling, the lead taproot of each plant (see Materials and methods) was isolated, imaged, and analyzed. The taproots of the HiNH_4_^+^ and LoNH_4_^+^ plants demonstrated considerable differences, as qualitatively indicated in **Figure 5**. In average, the lead taproots of HiNH_4_^+^ were shorter (55% reduction in total length, including both mother root and lateral roots), thinner (15% reduction in average diameter) and less branched (57% less forks) than those of the LoNH_4_^+^ plants.

#### 3.3.3 Leaf chlorophyll content, color index and F_v_/F_m_ at harvest

Dark-adapted chlorophyll fluorescence measurements of the maximum quantum yield of photosystem II (F_v_/F_m_) were identical for both treatments (**Table 3**), while an elevated SPAD index indicated a higher chlorophyll content of the leaves of the HiNH_4_^+^ plants. Color measurements illustrated significant differences as evaluated by the L*a*b color space, with the HiNH_4_^+^ leaf color shifted more towards green compared to the color of the LoNH_4_^+^ leaves.

**Table 3:** F_v_/F_m_ (average of two measurements per plant), SPAD (average of ten measurements per plant) and Color index (average of three measurements per plant) values of the 18 LoNH_4_^+^ and 18 HiNH_4_^+^ plants as measured at harvest (28 DAT).

Parameter	LoNH_4_^+^	HiNH_4_^+^	Statistical significance
F_v_/F_m_	0.83 ± 0.01	0.83 ± 0.01	*Not sign.*
SPAD index	20 ± 3	23 ± 2	p < 0.01
Color index L	53 ± 3	51 ± 3	*Not sign.*
Color index a	-18.8 ± 0.5	-18.3 ± 0.5	p < 0.01
Color index b	34.3 ± 1.3	32.6 ± 1.8	p < 0.01

All values are tabulated as mean value ± standard deviation. Statistical significance between the two treatments is indicated as p-values.

#### 3.3.4 Nutrient ion and element composition of shoots and roots at harvest

Nutrient ion and element analyses of lettuce shoot and root biomass harvested at 28 DAT demonstrated considerable differences between plants cultivated in the two different nutrient solutions (**Table 4**). The biomass content of NO_3_^-^ and NH_4_^+^ varied in line with their concentrations and ratios in the nutrient solutions. The HiNH_4_^+^ plants exhibited an NH_4_^+^ root content nearly three times higher and a NO_3_^-^ shoot and root content 7 – 8 times lower than that of the LoNH_4_^+^ plants. Considering total N, the most significant differences were observed for the roots, for which the total N content was 28% higher in the HiNH_4_^+^ plants. Additionally, the C content of HiNH_4_^+^ plants was higher than that of the LoNH_4_^+^ plants (9% higher in shoot content and 15% higher in root content). The content of other nutrient ions was generally lower in plants cultivated in HiNH_4_^+^ nutrient solution compared to those cultivated in LoNH_4_^+^. This includes the cations K^+^ (52% lower shoot content and 36% lower root content), Na^+^ (32% lower shoot content and 22% lower root content), and Mg^2+^ (47% lower shoot content). Ca^2+^ illustrated the same trend (55% lower shoot content and 17% lower root content in HiNH_4_^+^) although the differences were not statistically significant due to high variability in the results. Reduced nutrient ion content of HiNH_4_^+^ plants could also be observed for the anions HPO_4_^2-^ (44% lower shoot content) and Cl^-^ (72% lower root content), while no statistically significant differences could be observed for SO_4_^2-^.

**Table 4:** Average shoot and root nutrient ion and element content of the 18 LoNH_4_^+^ and 18 HiNH_4_^+^ lettuce plants as measured at harvest (28 DAT).

		LoNH_4_^+^	HiNH_4_^+^	Statistical significance
Shoots	Total C	382 ± 11	416 ± 9	p < 0.01
	Total N	39 ± 7	34 ± 5	p < 0.05
	NO_3_-N	6.0 ± 2.6	0.8 ± 0.3	p < 0.01
	NH_4_-N	1.0 ± 0.3	1.1 ± 0.2	*Not sign.*
	PO_4_-P	4.8 ± 1.1	2.7 ± 0.5	p < 0.01
	K^+^	71 ± 15	34 ± 6	p < 0.01
	Ca^2+^	3.8 ± 0.7	1.7 ± 0.3	*Not sign.*
	Mg^2+^	1.9 ± 0.3	1.00 ± 0.14	p < 0.01
	SO_4_-S	1.1 ± 0.3	1.3 ± 0.3	*Not sign.*
	Na^+^	4.6 ± 0.6	3.1 ± 1.4	p < 0.01
	Cl^-^	20 ± 5	17 ± 3	*Not sign.*
Roots	Total C	335 ± 19	384 ± 6	p < 0.01
	Total N	56 ± 2	72 ± 5	p < 0.01
	NO_3_-N	18 ± 3	2.7 ± 0.6	p < 0.01
	NH_4_-N	1.3 ± 0.2	3.5 ± 0.3	p < 0.01
	PO_4_-P	13.9 ± 1.0	15.2 ± 1.4	*Not sign.*
	K^+^	97 ± 11	62 ± 4	p < 0.01
	Ca^2+^	1.03 ± 0.17	0.9 ± 0.2	*Not sign.*
	Mg^2+^	1.07 ± 0.13	1.20 ± 0.12	*Not sign.*
	SO_4_-S	8.8 ± 1.7	9.4 ± 0.7	*Not sign.*
	Na^+^	4.7 ± 0.8	3.7 ± 1.0	p < 0.01
	Cl^-^	6.7 ± 1.4	1.9 ± 0.6	p < 0.01

All values are tabulated as mean value ± standard deviation in unit g/kg dry weight based on separate analyses of individual plants. Statistical significance between the two treatments is indicated as p-values.

### 3.4 Crop performance and system dynamics at high and low NH_4_^+^:NO_3_^-^ ratios

#### 3.4.1 O_2_ production

**Figure 6:**
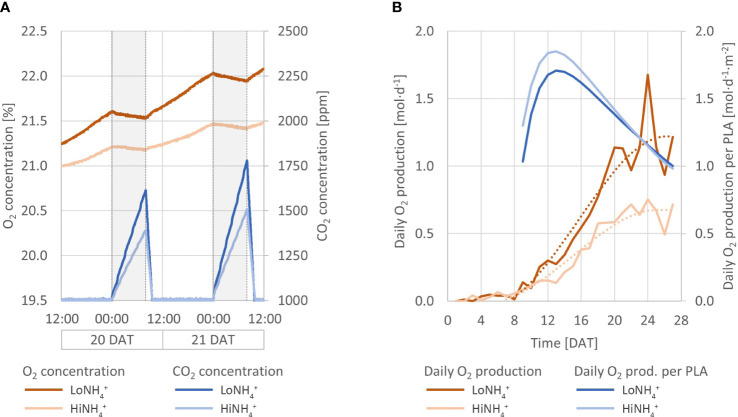
**(A)** Atmospheric O_2_ (red lines) and CO_2_ (blue lines) concentration inside the PCU cultivation chamber over 48 hours (20 - 21 DAT) for the LoNH_4_^+^ and HiNH_4_^+^ crop tests. Night phases are indicated with gray background. **(B)** Daily O_2_ production (by 18 plants; solid red lines) of the LoNH_4_^+^ and HiNH_4_^+^ crop tests, plotted together with smoothed datasets based on 3^rd^ degree polynomial regression (dotted red lines). Additionally, daily O_2_ production per projected leaf area (PLA; blue lines).

Continuous monitoring of the atmospheric O_2_ and CO_2_ concentrations inside the cultivation chamber illustrated O_2_ production during the day (when CO_2_ was controlled at 1000 ppm by automatic CO_2_ injection), and O_2_ consumption and CO_2_ production during the night (**Figure 2**). Detailed inspection of the datasets illustrates an immediate increase in atmospheric CO_2_ concentration and a corresponding decrease in O_2_ starting from the time at which the cultivation chamber LEDs were turned off, as illustrated in **Figure 6A**, detailing the first two days after the chamber venting. Reversely, an immediate decrease of CO_2_ concentration and increase of O_2_ was initiated when the LEDs were reactivated at the end of the night phase. During the 16-hour day phase of the 21^st^ day (20 DAT), the O_2_ concentration of the cultivation chamber increased 0.48 vol-% in the LoNH_4_^+^ crop test, and 0.26 vol-% in the HiNH_4_^+^ crop test. During this day, the LoNH_4_^+^ plants covered a 0.71 m^2^ cultivation area, while the HiNH_4_ plants covered 0.40 m^2^ (as evaluated by projected leaf area). During the following 8-hour night phase, the CO_2_ concentration increased linearly to 1614 ppm (LoNH_4_^+^) and 1391 ppm (HiNH_4_^+^) (with no injection of CO_2_). After reactivating the LEDs, the atmospheric CO_2_ concentration returned to 1000 ppm after 1.5 hours (LoNH_4_^+^) and 1.6 hours (HiNH_4_^+^) of daylight. Corresponding analyses at the end of the crop tests demonstrated an increase in O_2_ concentration of 0.59 vol-% by the LoNH_4_^+^ plants (1.21 m^2^ projected leaf area), and 0.33 vol-% by the HiNH_4_^+^ plants (0.71 m^2^ projected leaf area) during the day phase of the 28^th^ day. The total O_2_ amount inside the PCU atmospheric loop and its pressure compensation system was precisely determined at the time of cultivation chamber closing and opening (see Materials and methods). Compensating for the accurately determined chamber gas leaks, this allows detailed calculations of the O_2_ amount produced by the plants during the two phases of each crop test before (0 - 18 DAT) and after (20 - 28 DAT) chamber venting (totaling 26.6 days; **Table 5**). Considering the absolute O_2_ production, the HiNH_4_^+^ plants produced 34% less O_2_ during the first phase (0 - 18 DAT) and 43% less O_2_ during the second phase (20 - 28 DAT) compared to the LoNH_4_^+^ plants. However, accounting for the smaller projected leaf area (PLA) of the HiNH_4_^+^ plants, average O_2_ production per day and projected leaf area was similar for the LoNH_4_^+^ and HiNH_4_^+^ plants both during the first phase of the crop tests (1.2 vs 1.3 mol·d^-1^·m^-2^, respectively) and during the second phase (both 1.1 mol·d^-1^·m^-2^). Furthermore, relating the total O_2_ production to the total shoot DW at harvest also demonstrates similar values for the two crops, with 37 mol O_2_ produced (over 26.6 days) per kg shoot DW for both crops. Estimated daily O_2_ production throughout the crop tests was calculated based on the O_2_ concentration in the cultivation chamber, the leak rate, and estimated daily gas composition of the atmospheric pressure compensation system (see Materials and methods). As the latter introduces a modest uncertainty in the calculations of daily O_2_ production, the resulting datasets were smoothed by polynomial regression (**Figure 6B**). Evaluating the differences in daily O_2_ production at 10, 14, 18, 22 and 26 DAT based on these datasets, the HiNH_4_^+^ plants produced 26, 37, 40, 42 and 45% less compared to the LoNH_4_^+^ plants, respectively.

**Table 5:** Total O_2_ production of 18 plants during the LoNH_4_^+^ and HiNH_4_^+^ crop tests, tabulated for the cultivation periods before (18.1 days) and after (8.5 days) the chamber venting, in addition to the full crop test period (26.6 days, not accounting for the day of venting at 19 DAT).

	Phase (duration)	LoNH_4_^+^	HiNH_4_^+^
Total O_2_ production [mol]	0 - 18 DAT (18.1 days)	3.61	2.40
Average daily O_2_ production [mol·d^-1^]		0.20	0.13
Average PLA [m^2^]		0.16	0.10
PLA-specific daily O_2_ production [mol·d^-1^·m^-2^]		1.22	1.32
Total O_2_ production [mol]	20 - 28 DAT (8.5 days)	8.74	4.97
Average daily O_2_ production [mol·d^-1^]		1.03	0.59
Average PLA [m^2^]		0.97	0.55
PLA-specific daily O_2_ production [mol·d^-1^·m^-2^]		1.06	1.06
Total O_2_ production (26.6 days) [mol]	(26.6 days)	12.4	7.37

Average daily O_2_ production and average projected leaf area (PLA) are calculated based on the tabulated period durations, as a basis for calculation of PLA-specific daily O_2_ production.

To account for the considerable difference in biomass between the two treatments, the daily O_2_ production (smoothed dataset) was evaluated per projected leaf area (PLA), measured non-destructively throughout the crop tests. These results (**Figure 6B**), illustrate similar daily O_2_ productions per PLA for both treatments, with an indication of an increased O_2_ production per PLA for the HiNH_4_^+^ plants during the early phase of the crop tests (15, 7, and 4% increase at 10, 14 and 18 DAT, respectively, and no difference from 20 to 28 DAT). The highest specific O_2_ productivity (per time and PLA) estimated was 1.85 mol·d^-1^·m^-2^ for the HiNH_4_^+^ crop (13 DAT) and 1.71 mol·d^-1^·m^-2^ for the LoNH_4_^+^ crop (13 DAT).

#### 3.4.2 Nutrient solution development

From start, the total N concentration was the same in both crop tests (11 mM; **Table 1**), while the NH_4_^+^:N ratios were different (0.1 mol·mol^-1^ for LoNH_4_^+^, 0.5 mol·mol^-1^ for HiNH_4_^+^). Based on an advanced PCU nutrient management system, automatic feeding of nutrient stock solutions (with the same NH_4_^+^:N ratios as from start, thus 0.1 or 0.5 mol·mol^-1^, see Materials and methods) successfully maintained a constant NO_3_^-^ concentration throughout both crop tests (**Figure 3A**), at 9.7 mM for LoNH_4_^+^ and 5.6 mM for HiNH_4_^+^ (**Table 1**). While this control strategy required considerable feeding of nutrient stock solutions during the LoNH_4_^+^ crop test (0.5 L of each stock solution), practically no nutrient stock solution feeding was required during the HiNH_4_^+^ crop test (0.03 L of each), indicating a considerable NO_3_^-^ consumption during the LoNH_4_^+^ crop test, and practically no NO_3_^-^ consumption during the HiNH_4_^+^ crop test.

Without control of nutrient solution NH_4_^+^ concentration, NH_4_^+^ levels were allowed to vary based on N consumption. Throughout both crop tests, the NH_4_^+^ concentration was considerably reduced (**Table 1**), demonstrating a higher consumption of NH_4_^+^ relative to total N than the NH_4_^+^:N ratio of the nutrient solution. In consequence, the nutrient solution NH_4_^+^:N ratio during the LoNH_4_^+^ crop test was reduced from 0.12 to 0.02 mol·mol^-1^, while it was reduced from 0.49 to 0.20 mol·mol^-1^ during the HiNH_4_^+^ crop test.

Nitrite (NO_2_^-^) could not be detected (< 1 mg N L^-1^) throughout the full LoNH_4_^+^ crop test and throughout the close-to-exponential phase of the HiNH_4_^+^ crop test (up to 20 DAT). During the latter test, NO_2_^-^ was detected during the last 6 days of cultivation (0.1 mM at 22 DAT, increasing to 0.5 mM at 28 DAT). To rule out major effects of nitrification on observed NH_4_^+^ consumption, operation of the cultivation facility was prolonged after removal of all plants from the system. Over the next 6 days, the NH_4_^+^ concentration decreased 0.5 mM in parallel with a further 0.4 mM increase of NO_2_^-^. Nutrient solution pH was kept constant by automatic pH control with KOH and H_2_SO_4_. No acid addition was required in any of the crop tests, while base addition was characterized by a considerably higher KOH consumption during the HiNH_4_^+^ crop test (1.6 mol OH^-^) than during the LoNH_4_^+^ crop test (0.3 mol OH^-^) (**Figure 3B**).

Despite no control of electrical conductivity (EC), the NO_3_^–^based feeding of nutrient stock solutions and the pH-based feeding of base resulted in a relatively constant EC of 1.9 mS/cm during the LoNH_4_^+^ crop test and 2.2 - 2.3 mS/cm during the HiNH_4_^+^ crop test (**Table 1**).

Development of macronutrient concentrations (**Figure 2B**) in the nutrient solution was most profound for K^+^, with a considerable increase from 3.5 to 7.4 mM during the HiNH_4_^+^ crop test (KOH was automatically added to maintain pH at setpoint). In contrast, K^+^ concentration decreased by 0.6 mM over the LoNH_4_^+^ crop test. Other ions demonstrating a concentration change of more than 0.2 mM over the course of a crop test (beyond K^+^ and N ion species) included Ca^2+^, Na^+^ and Cl^-^, demonstrating an increase during the LoNH_4_^+^ crop test (requiring considerable feeding of nutrient stock solutions) and a decrease during the HiNH_4_^+^ (conducted practically without feeding of nutrient stock solutions). For other macronutrients, including HPO_4_^2-^, Mg^2+^, and SO_4_^2-^, analyses throughout the crop tests demonstrated stable levels (**Table 1**).

#### 3.4.3 N mass balance

With a considerable nutrient solution volume (270 L), a limited cultivation area (1.8 m^2^), and a crop with modest nutrient requirements (lettuce), mass balances are vulnerable to measurement inaccuracies especially with respect to ion concentrations in the nutrient solution. Nevertheless, a N mass balance was calculated (**Table 6**) based on the available datasets, demonstrating a mass balance closure of 94% (LoNH_4_^+^) and 89% (HiNH_4_^+^).

**Table 6:** Nitrogen (N) mass balance of the LoNH_4_^+^ and HiNH_4_^+^ crop tests.

	Parameter and unit	LoNH_4_^+^	HiNH_4_^+^
Nutrient solution	NO_3_^-^ at start [mol]	2.66	1.47
	NH_4_^+^ at start [mol]	0.36	1.39
	Total inorganic N at start [mol]	3.02	2.87
	NO_3_^-^ addition [mol]	0.82	0.03
	NH_4_^+^ addition [mol]	0.10	0.03
	Total inorganic N net addition [mol]	0.90	0.05
	NO_3_^-^ at end [mol]	2.58	1.54
	NH_4_^+^ at end [mol]	0.07	0.43
	NO_2_^-^ at end [mol]	0.00	0.14
	Total inorganic N at end [mol]	2.65	2.12
	Total inorganic N consumption [mol]	1.28	0.80
Plants	N in shoots [mol]	1.02	0.53
	N in roots [mol]	0.17	0.18
	N in biomass [mol]	1.19	0.70
Mass balance	Mass balance closure [%]	94	89

Total inorganic N net addition accounts for addition of inorganic N (as NO_3_^-^ and NH_4_^+^) and samples removed throughout the crop tests. Mass balance closure is calculated as the amount of N in the total biomass divided by the total consumption of inorganic N.

The total consumption of NO_3_^-^ and NH_4_^+^ during the LoNH_4_^+^ crop test was 0.9 and 0.4 mol, respectively, illustrating an apparent NH_4_^+^:N consumption ratio of 0.31 mol·mol^-1^ during the LoNH_4_^+^ crop test. In contrast, with no observed consumption of NO_3_^-^ during the HiNH_4_^+^ crop test, N consumption was apparently based on NH_4_^+^ only. Relative to the produced biomass (**Table 2**), the consumption of inorganic N (**Table 6**) was 3.1 mol·kgDW^-1^ for both crop tests.

#### 3.4.4 Incorporation of C and N into edible biomass

**Figure 7:**
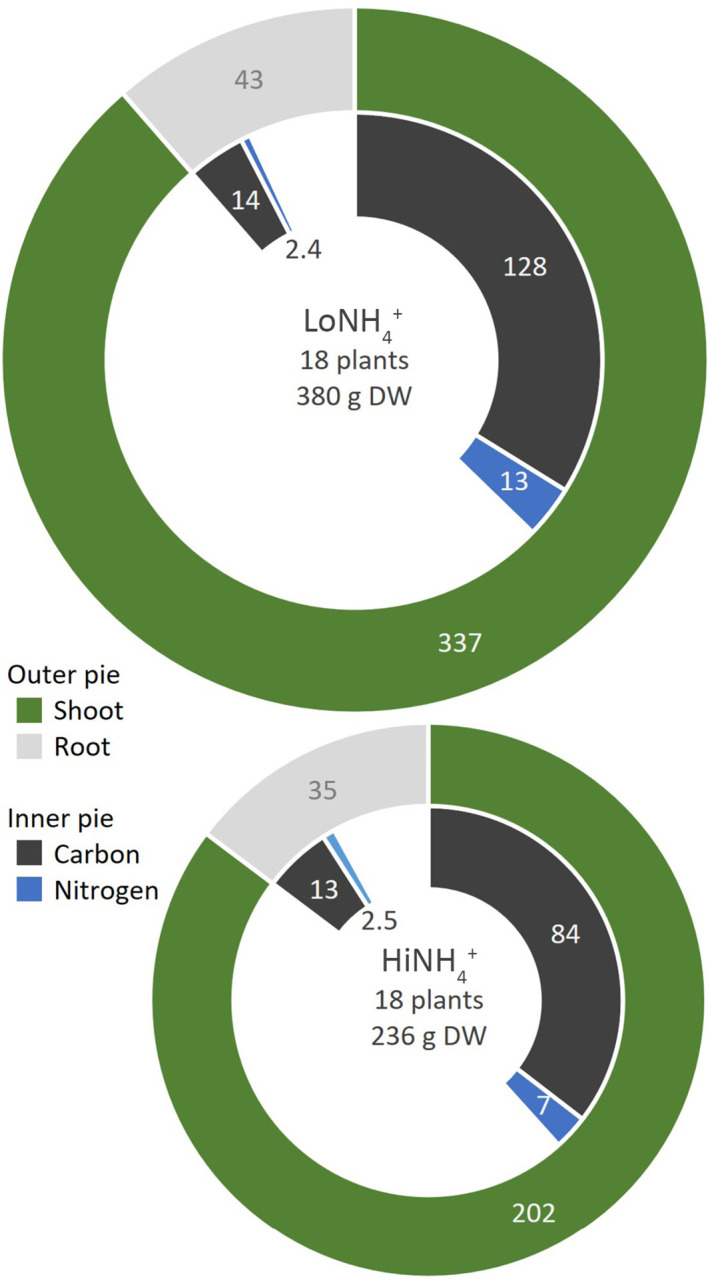
Incorporation of C (black) and N (blue) into shoots (green) and roots (gray) of the LoNH_4_^+^ (upper) and HiNH_4_^+^ (lower) crops. All numbers represent element or biomass dry weight in gram and relate to the full crop consisting of 18 plants. The relative difference in total pie area corresponds to the relative difference in total dry weight of the two crops.

To illustrate C and N recycling in a BLSS perspective, the amounts of C and N incorporated into edible biomass (18 plants per crop) was calculated for both crop tests (**Figure 7**) based on measured shoot DW at harvest (**Table 2**), and their composition (**Table 4**). The edible biomass of the HiNH_4_^+^ crop (202 g DW) was 40% lower than that of the LoNH_4_^+^ crop (337 g DW), while the relative shoot C content of the HiNH_4_^+^ crop (42%) was higher than that of the LoNH_4_^+^ crop (38%). In sum, the HiNH_4_^+^ crop incorporated 35% less C (84 g) into total edible biomass compared to the LoNH_4_^+^ crop (128 g). For N, the relative shoot content of the HiNH_4_^+^ crop (3.4%) was lower than that of the LoNH_4_^+^ crop (3.9%). In sum, the HiNH_4_^+^ crop incorporated 48% less N (7 g) into total edible biomass compared to the LoNH_4_^+^ crop (13 g).

## 4 Discussion

During long duration human space missions, bioregenerative life support systems may provide food, regenerated water, and O_2_ for the astronauts. Given the limited availability of nutrient resources for plant production in this scenario, recycling and reuse of nutrients such as N from human urine is important. Depending on the upstream mineralization process of urine, the nutrient solution may have various NH_4_^+^:NO_3_^-^ ratios and elevated NaCl concentrations. To maximize production of food, O_2_ and clean water, crop cultivation conditions should be optimized. However, as a BLSS consists of multiple processes, the performance of the regenerative loop including ammonification and nitrification needs to be evaluated and optimized as a whole. This illustrates a possible trade-off between the overall BLSS efficiency and crop cultivation and yield, which should be considered in a long-term perspective with continuous operation. Increased knowledge on plant responses to various optimal and sub-optimal cultivation conditions and nutrient solutions will contribute to improved computer models, BLSS efficiency and improvement in design and control of integrated processes linking waste management and crop cultivation.

### 4.1 Biomass production and morphology

In this research campaign, considerable differences were identified between lettuce plants cultivated in nutrient solutions containing high and low NH_4_^+^:N ratios. In a BLSS perspective, the results demonstrate that both NH_4_^+^:N ratios, representing considerably different scenarios of upstream waste treatment, allow plant growth and development, and thus fixation of C and N into edible biomass. There is, however, a clear impact on biomass productivity (produced amount per time) and therefore on the total production over a given time. Under the conditions tested during the 28-day crop tests performed in this campaign, the HiNH_4_^+^ crop provided 40% lower edible biomass (shoot dry weight), resulting in the incorporation of 35% less C and 48% less N into edible biomass compared to the crop cultivated at low NH_4_^+^:N. These results are in line with previous research on lettuce, illustrating that increased ammonium concentrations strongly affect growth, resulting in decreased root and shoot biomass accumulation (Qiu et al., 2014; Wenceslau et al., 2021; Du et al., 2022; Hameed et al., 2022). Furthermore, the results are in line with general effects of ammonium toxicity on plant growth, demonstrating a biomass-reducing effect of high ammonium concentrations attributed to for example oxidative stress, nutrient imbalances and reduced photosynthesis (Barickman and Kopsell, 2016; Song et al., 2021; Weil et al., 2021). In line with the biomass results, the total leaf area per plant at time of harvest was reduced by a 2-factor for the HiNH_4_^+^ plants compared to the LoNH_4_^+^ plants. This effect is supported by other reports on hydroponically grown lettuce and other plants, demonstrating lower leaf areas with increasing ammonium concentrations (Guo et al., 2007a; Guo et al., 2007b; Urlic et al., 2017; Wenceslau et al., 2021). Beyond the NH_4_^+^:N ratio, other potential stress factors include the nutrient solution’s total ionic strength and the presence of Na^+^ and Cl^-^ added to mimic a urine recycling scenario. While several studies indicate no or minor growth retardation of lettuce cultivated in the presence of seawater, elevated electrical conductivity and/or Na^+^ and Cl^-^ concentrations similar to the cultivation conditions used in the presented work (Atzori et al., 2019 and references within), one should not rule out combined effects of salinity and NH_4_^+^:N ratio especially in a foreseen future closed-loop scenario with accumulating Na^+^ and Cl^-^ concentrations and increasing total electrical conductivity.

Calculation of PLAI throughout the crop tests offered a method to evaluate plant growth and PLAI-based growth rate without opening the closed cultivation chamber. While noticeable differences in PLAI (and thus biomass) between the HiNH_4_^+^ and LoNH_4_^+^ crops could not easily be observed until after approximately 10 days of cultivation, calculation of µ_PLAI_ indicated a difference in specific growth rate between the two crops starting already in the early cultivation phase. This indicates that under the conditions tested, the elevated NH_4_^+^:N ratio introduced negative effects on plant growth also during the close-to-exponential cultivation period. At the same time, this indicates that the observed NO_2_^-^ in the nutrient solution was not the main cause of the observed differences in biomass production (and thus O_2_ production, see below). While elevated NO_2_^-^ concentrations in the nutrient solution may cause reduced growth (Hoque et al., 2008), NO_2_^-^ was not observed until the late cultivation phase of the HiNH_4_^+^ crop, at a time which µ_PLAI_ of both crops had started to decrease and coincided. Coinciding µ_PLAI_ towards the end of the cultivation period may be explained by reduced specific growth rates as plants mature, and the fact that the presented growth rates are based on PLAI which, by definition, cannot exceed a factor of 1 due to a limited cultivation area and overlapping leaves.

Beyond the overall reduction in biomass production of the HiNH_4_^+^ crop, these plants displayed a significantly higher root:shoot ratio than the LoNH_4_^+^ plants, indicating a higher relative use of energy towards root growth. This is in line with previous research showing increasing root:shoot ratio with increasing NH_4_^+^:NO_3_^-^ ratio in several species, including lettuce (Zhu et al., 2020), tobacco (Walch-Liu et al., 2000) and cucumber (Zhou et al., 2017). More specifically, alteration of biomass partitioning between root and shoot is a known plant response to nutritional stress (Maskova and Herben, 2018) and NH_4_^+^:K^+^ imbalance has been demonstrated to negatively influence carbohydrate accumulation, disturbing the root-to-shoot biomass partitioning (Zhao et al., 2020). In our study, NH_4_^+^ and K^+^ were among the nutrient ions that demonstrated the greatest differences between the biomass content of the HiNH_4_^+^ and LoNH_4_^+^ crops. Additionally, the two crops demonstrated differences in root size and morphology. The HiNH_4_^+^ plants exhibited shorter primary and secondary roots, along with a significantly lower number of forks and links illustrating less branching. This is likely to be caused by the nutrient availability (especially of NH_4_^+^ and NO_3_^-^) and plant nutritional status, which are known to strongly influence the development of the root system (Lima et al., 2010). NH_4_^+^ is known to affect root architecture by inhibition of primary root growth and elongation, while promoting lateral branching (Liu and Von Wiren, 2017; Liu et al., 2013). Conversely, NO_3_^-^ has been shown to stimulate lateral root elongation (Lima et al., 2010; Marschner and Marschner, 2012). These observations represent valuable details for mathematical modelling of plant growth and development, as the root’s surface area is critical for mass and energy transfer.

### 4.2 O_2_ production

In total, the HiNH_4_^+^ crop produced 41% less O_2_ than the LoNH_4_^+^. However, as the growth of the HiNH_4_^+^ plants was impaired, the O_2_ production should be evaluated also in terms of specific production. When O_2_ production was normalized by shoot dry weight at harvest, the amount of O_2_ produced was 37 mol·kg^-1^ for both the LoNH_4_^+^ and the HiNH_4_^+^ crops. On a high level, O_2_ production following biomass production is in line with the general mechanism of photosynthesis, converting light energy to chemical energy utilized for the synthesis and accumulation of organic matter (Evert and Eichhorn, 2013; Taiz, 2015), and hence, the photosynthetic rate is determining for biomass productivity. Furthermore, measurements of chlorophyll fluorescence on dark-adapted leaves demonstrated that the F_v_/F_m_ of both crops (0.83) was within a range indicating a healthy and well-functioning photosystem II (0.79 – 0.84) (Bjorkman and Demmig, 1987).

The total O_2_ production, when normalized per unit leaf area at harvest, was 1.8 and 2.1 mol·m^-2^ for the LoNH_4_^+^ and HiNH_4_^+^ crops, respectively. Similarly, O_2_ production per PLA demonstrates no reduction in specific O_2_ production of the HiNH_4_^+^ plants compared to the LoNH_4_^+^ plants. On the contrary, detailed examination of the data indicates a marginal increase of the specific O_2_ production per PLA by the HiNH_4_^+^ plants during the second and third week of the cultivation period. This indication is in line with a higher SPAD index of the leaves of the HiNH_4_^+^ plants, and a color index more towards green compared to the LoNH_4_^+^ plants. These findings are again in line with other investigations on both lettuce and species such as Arabidopsis and kohlrabi, demonstrating a higher chlorophyll concentration in leaves of plants cultivated with a higher proportion of NH_4_^+^ in the fertilizer (Blanke et al., 1996; Qiu et al., 2014; Hu et al., 2015). However, the scientific literature on chlorophyll content as a response to NH_4_^+^ concentration and NH_4_^+^:NO_3_^-^ ratio demonstrate complex relationships and varying results. Song et al. (2021) demonstrated that lettuce and cabbage chlorophyll content was higher in plants exposed to an NH_4_^+^:NO_3_^-^ ratio of 50:50, than both 0:100 and 100:0, while Zhu et al. (2020) reported a higher chlorophyll concentration in lettuce seedlings exposed to an NH_4_^+^:NO_3_^-^ ratio 25:75 than both 100:0, 50:50 and 0:100. Studies of other species also show varying results, for example, an increase in chlorophyll content with increasing NH_4_^+^:NO_3_^-^ ratios was not discovered in kale (Assimakopoulou et al., 2019). Different theories are proposed to explain an increased chlorophyll content as a response to high ammonium. The higher chlorophyll concentration may be caused by ammonium-stress inhibiting leaf expansion, resulting in a denser chlorophyll content rather than an actual total increase (Sanchez-Zabala et al., 2015). Furthermore, carbon skeletons in the leaves favor NH_4_^+^ assimilation. Based on this, it has been proposed that a higher chlorophyll content might be a strategy to increase photosynthetic CO_2_ assimilation to produce more carbon skeletons and thereby mitigate NH_4_^+^ accumulation (Sanchez-Zabala et al., 2015).

### 4.3 Biomass composition

The 3-fold increase of root NH_4_^+^ content of the HiNH_4_^+^ plants compared to the LoNH_4_^+^ plants is comparable to the difference between the two nutrient solutions, with 3 to 5-fold elevated NH_4_^+^ concentration (at start and end, respectively) and 3 to 7-fold elevated NH_4_^+^:N ratio in the HiNH_4_^+^ nutrient solution compared to the LoNH_4_^+^ nutrient solution. Such elevated root NH_4_^+^ content is consistent with the roots being the primary site for initial NH_4_^+^ accumulation, where it is incorporated into organic complexes, while N is typically transported to the shoots in other forms such as amino acids (Mengel and Kirkby, 2001; Marschner and Marschner, 2012). Additionally, the biomass NO_3_^-^ content varied considerably between the crops, and in line with the nutrient solution composition. The shoots and roots of the HiNH_4_^+^ plants demonstrated an 87% and 85% reduction compared to those of the LoNH_4_^+^ plants, respectively, in line with the 42% lower NO_3_^-^ concentration in the nutrient solution. A similar reduction of leaf NO_3_^-^ content with increasing NH_4_^+^:N ratio in the nutrient solution was reported for example for rocket salad (Kim et al., 2006). The average nitrate content of the LoNH_4_^+^ and HiNH_4_^+^ edible biomass (shoots) was 6.0 and 0.8 g NO_3_-N per kg dry weight, respectively (**Table 4**). Considering the shoot dry weight content (**Table 2**), this corresponds to 1.8 g and 0.3 g NO_3_^-^ per kg fresh weight, respectively, both below the maximum nitrate levels set by the European Union (Commission Regulation No 1258/2011).

Analyses of the shoot and root content of nutrient ions beyond N species demonstrated an overall trend of similar or lower content of both anions and cations in the HiNH_4_^+^ plants compared to the LoNH_4_^+^ plants. As demonstrated by the ionic concentration trend of the nutrient solution, all macronutrients beyond NH_4_^+^ were available at relatively stable concentrations throughout the crop tests, indicating that the differences between the two crops were not governed by nutrient availability, but rather by direct or indirect consequences of the difference in NH_4_^+^ concentration and/or NH_4_^+^:N ratio. This is in line with reports on reduced uptake of ions such as K^+^, Ca^2+^ and Mg^2+^ with increasing NH_4_^+^ concentration and NH_4_^+^:NO_3_^-^ ratio in the nutrient solution (Britto and Kronzucker, 2002; Savvas et al., 2006; Roosta and Schjoerring, 2008; Fallovo et al., 2009). For example, Weil et al. (2021) reported that the concentration of both K^+^ and Ca^2+^ in lettuce shoots declined significantly with increasing nutrient solution NH_4_^+^:N ratio, concluding that the uptake of cationic nutrients and the plant growth were reduced when the NH_4_^+^:N ratio exceeded 0.50 mol·mol^-1^. The observed effects on nutrient ion content have been explained by antagonism (Lasa et al., 2001; Marschner and Marschner, 2012); that the NH_4_^+^ ion resembles the K^+^ ion in radius and hydration shell size and may therefore move through K^+^ channels (Marschner and Marschner, 2012), and that uptake of NO_3_^-^ occurs simultaneously with the uptake of Ca^2+^ or K^+^ so that an increasing proportion of NO_3_^-^ will increase the content of Ca^2+^ and K^+^ in leaves (Du et al., 2022).

The content of NaCl in the nutrient solutions, which was designed to mimic a urine recycling scenario, may have impacted plant nutrient uptake in both the high and low NH_4_^+^- scenarios. In general, excess Na^+^ uptake lowers the uptake of essential ions and disturbs the root-shoot transport. It has been widely observed that Na^+^, even at low levels, inhibits the transport systems for K^+^ uptake due to the chemical similarities between the two ions (Amini and Ehsanpour, 2005; Kronzucker et al., 2013). For some functions, Na^+^ may also replace potassium, such as for example for maintenance of vacuole osmotic potential (Wakeel et al., 2011). Na^+^ may cause osmotic stress, which decreases the passive uptake of calcium, resulting in a lower concentration of calcium in the plant. Another possible effect of NaCl in plant nutrition, is that Cl^-^ may antagonize the uptake of nitrate, while it assists uptake of ammonium (Song et al., 2021).

The HiNH_4_^+^ lettuce crop demonstrated a higher carbon content in both leaves and roots compared to the LoNH_4_^+^ plants. During stress conditions that affect plant growth but still allow for photosynthesis, accumulation of sugars is commonly observed Peshev and Van Den Ende, 2013). Exposed to high NH_4_^+^ concentrations, plants have been shown to synthesize sugars to provide carbon skeletons to increase NH_4_^+^ assimilation (Raab and Terry, 1994). Additionally, plants exposed to abiotic stress often accumulate reactive oxygen species (ROS). Accumulation of soluble sugars can mitigate this toxic effect caused by plant stress by contributing to ROS scavenging (Keunen et al., 2013; Peshev and Van Den Ende, 2013). Sugars can also protect chloroplasts and thereby stabilize photosynthesis under stress conditions, and they may serve as osmoprotectants ( Peshev and Van Den Ende, 2013). Additionally, the uptake of NO_3_^-^ against the electrochemical gradient requires more use of fixed carbon compared to acquisition of NH_4_^+^ (Britto and Kronzucker, 2013).

### 4.4 Nutrient consumption and nutrient solution evolution in a long-term perspective

Maximizing resource utilization limits the possibilities of flushing the hydroponic loop and restarting the system with a fresh and balanced nutrient solution. Thus, long term development of the nutrient solution needs to be understood, modelled, and ultimately designed and controlled in a trade off with the productivity of the plants and the design and performance of upstream processes. In light of the observed effects of the NH_4_^+^:N ratio, the development of this ratio over time should be considered. The results obtained under the conditions tested indicate a preference for NH_4_^+^ during the HiNH_4_^+^ crop test, in which N consumption apparently almost exclusively could be attributed to NH_4_^+^ consumption, although one cannot rule out that a modest nitrification activity could convert some NH_4_^+^ to NO_3_^-^ and thus mask some NO_3_^-^ consumption by the plants. Also, during the LoNH_4_^+^ crop test, the consumption of NH_4_^+^ relative to total N in the system was higher than the concentration of NH_4_^+^ relative to total N in the nutrient solution. In consequence, the nutrient solution NH_4_^+^:N ratio decreased over time during both crop tests (from 0.12 to 0.02 mol·mol^-1^ in the LoNH_4_^+^ crop test; from 0.49 to 0.20 in the HiNH^+^_4_ crop test). Even after a hypothetical addition of nutrient stock solutions to restore the total N concentration before an additional plant cultivation period, the initial NH_4_^+^:N ratio of the second period will be lower than that of the first (0.03 mol·mol^-1^ after topping up the remaining LoNH_4_^+^ nutrient solution to the initial 11 mM total N; 0.29 mol·mol^-1^ after topping up the remaining HiNH_4_^+^ nutrient solution to the initial 11 mM total N). These considerations illustrate a potential to combine high-NH_4_^+^:N stock solutions generated by upstream waste treatment processes with efficient long-term plant biomass and O_2_ productivity despite initially reduced productivity. In a modelling perspective, it illustrates the importance of understanding the relative NH_4_^+^:NO_3_^-^ consumption in the higher plant compartment combined with the effects of the NH_4_^+^:NO_3_^-^ ratio of the mineralized waste from upstream compartments. High NH_4_^+^ to NO_3_^-^ preference has been observed in several other studies, including a study of N absorption by tomato, in which 50% of plant N was absorbed as NH_4_^+^⁠ even though this ion represented only 10% of available N (the remaining 90% being NO_3_^-^) (Glass et al., 2002). N preference may be linked to energy considerations as uptake of NO_3_^-^ requires energy while NH_4_^+^ can be directly absorbed, hence many plants prefer to take up NH_4_^+^ if both are available (Mengel and Kirkby, 2001; Marschner and Marschner, 2012; Du et al., 2022). Additionally, NO_3_^-^ uptake is inhibited by ammonium, and lettuce appears to absorb ammonium faster than NO_3_^-^ when the source contains both N-forms (Savvas et al., 2006; Britto and Kronzucker, 2013). However, plant preference for NH_4_^+^ or NO_3_^-^ is known to vary across plant species, physiological phase and environmental conditions (Britto and Kronzucker, 2013), and thus observed N preference may only be valid for a given species, developmental stage, and cultivation conditions. An improved understanding of the NH_4_^+^ to NO_3_^-^ preference, for example as function of cultivation conditions, should be further pursued, as this may hold potential to reduce ammonium toxicity even at conditions of high NH_4_^+^:N ratios.

During the LoNH_4_^+^ crop test which required a considerable amount of nutrient stock solution feeding, accumulation of Na^+^, Ca^2+^ and to a certain extent Cl^-^ was observed together with a marginal reduction of K^+^. This illustrates imbalance between the system’s feed rates and consumption rates. Nutrient ion levels in the nutrient stock solutions may be tuned relative to for example total N as far as charge balancing allows in order to achieve a balanced nutrient solution. Accumulation of Na^+^ and Cl^-^, however, represents a potential general challenge for the utilization of mineralized human waste. Accumulation of Na^+^ in the nutrient solution may to a certain extent be mitigated by increased plant Na^+^ uptake rates with increasing Na^+^ concentrations in the nutrient solution (Neocleous and Savvas, 2017; Breś et al., 2022). Nevertheless, the extent and the implications of long-term accumulation of non-nutrients in crop cultivation based on human waste are important aspects that would benefit from further attention in a BLSS perspective.

The ratio between consumed NH_4_^+^ and consumed NO_3_^-^ affects nutrient solution pH. NH_4_^+^ uptake causes release of protons and thereby reduced pH in the root zone, which again may impact nutrient uptake (Lasa et al., 2001; Marschner and Marschner, 2012; Weil et al., 2021). This effect was clearly demonstrated during the HiNH_4_^+^ crop test with a substantial amount of base added to maintain a constant pH. The HiNH_4_^+^ scenario, with an NH_4_^+^:N ratio of 0.5 mol·mol^-1^ is based on urine ammonification and partly nitrification without addition of alkalinity (Larsen et al., 2021). In a BLSS scenario, the results presented illustrate that no or low alkalinity addition during the nitrification step results in increased alkalinity requirement during the crop cultivation step instead. In this case, addition of OH^-^ during crop cultivation should be carefully balanced with appropriate anions to avoid nutrient imbalance over time. In this context, it is interesting to notice that addition of macronutrient ions prone to be present at low concentrations in mineralized urine due to precipitation (such as Ca^2+^) could possibly serve a dual purpose of being a counter ion for OH^-^ charge balancing and being a required nutrient solution supplement. Such feeding strategies must, however, be carefully tuned and consider factors such as salt availability and solubility.

### 4.5 Strategies to maximize food and O_2_ production in a BLSS perspective

In a Lunar greenhouse perspective, cultivation area will be limited and should be utilized in an efficient way. In a scenario of constant plant density throughout the cultivation period, plants should be cultivated to an adult stage, as the plant growth rate (g per day) and the O_2_ production rate (mol per day) are highest during the end of the cultivation period, thus increasing also the average growth rate and the average O_2_ production rate as evaluated over the full cultivation period. However, such a strategy comes at the cost of a low utilization rate of the available cultivation area (low PLAI), especially during the first part of the cultivation period. In a more optimized scenario, initial plant density can be high to achieve a high PLAI even with small plants. As the plants grow, the plant density can be reduced to give room for the expanding plants. In such an optimized scenario, specific production per time and PLA should be considered when aiming at optimizing food (biomass) and O_2_ production. Under the conditions tested, the specific O_2_ production per time and PLA reached a maximum around 13 DAT (**Figure 6B**), indicating that adolescent lettuce plants were more effective in O_2_ production per time and PLA than seedlings and adult plants. With O_2_ production linked to biomass production, this period with the highest specific O_2_ production per time and PLA coincides with the period of highest specific growth rate (here estimated as µ_PLAI_; **Figure 4**). Thus, in an optimized scenario with dynamic plant density to continuously operate at a high PLAI, the results obtained under the conditions tested suggest that plants should be cultivated up to approximately 16 - 20 days after transplant (26 - 30 days after germination) to maintain a maximized specific biomass (food) production per time and PLA and a maximized specific O_2_ production per time and PLA. Furthermore, it is interesting to notice the indication of a higher O_2_ production per time and PLA of the HiNH_4_^+^ crop compared to that of the LoNH_4_^+^ crop (**Figure 6B**), illustrating that as long as PLAI is kept high, O_2_ productivity may be high even under cultivation conditions suboptimal for growth. Such an effect, that may be attributed to the plant’s complex physiological response to mitigate suboptimal cultivation conditions, is especially interesting considering the possible need to accept suboptimal conditions for a given BLSS step in order to maximize the total efficiency of the whole loop.

## 5 Conclusion

The results of this study demonstrate the importance of understanding and considering direct and indirect effects of upstream waste processing on crop cultivation in BLSS. The urine utilization scenarios of 0.1 and 0.5 mol·mol^-1^ NH_4_^+^:N demonstrated significant effects on plant development, plant nutrient composition and O_2_ production. Under the conditions tested, plants cultivated at the high NH_4_^+^:N ratio demonstrated a high preference for ammonium combined with a reduced specific growth rate and thus a reduced total biomass production. O_2_ production per time and projected leaf area reached a maximum during the adolescent plant phase, coinciding with exponential growth and indicating a strong relationship between biomass and O_2_ production. Interestingly, the specific O_2_ production per time and projected leaf area was marginally higher for the plants cultivated at high NH_4_^+^:N ratio, in line with a higher chlorophyll content. In a life support perspective, the results illustrate design concepts for crop cultivation strategies depending on the performance of upstream processes (e.g. urine mineralization and high/low NH_4_^+^:N) and downstream needs (food or O_2_). In addition, parameters such as plant density and cultivation duration may be adjusted or balanced against each other to optimize performance. Ultimately, the results may guide the design and control of both crop cultivation and other processes in a regenerative BLSS loop towards a common trade-off and to achieve a greater good rather than optimizing individual processes.

The unique features of the ESA MELiSSA Plant Cultivation Unit (PCU) with its gas-tight atmospheric and hydroponic loops with extensive monitoring and control enabled collection of comprehensive and detailed data on crop O_2_ production, plant growth via image-based analyses, and nutrient solution dynamics throughout the entire cultivation period. Together with future expanded capabilities of the PCU, such as analyses of CO_2_ consumption and water production, this will contribute to improved understanding of plant responses and further advancement of computer models for predictions of plant growth and production in various BLSS and cultivation scenarios.

This study represents a further advancement towards increased waste- and resource recycling in plant-based food production systems. A deeper understanding of these processes and their impacts is crucial for the production of food and for the regeneration of water and O_2_ in future human space exploration. Additionally, considering relevant terrestrial challenges such as depletion of mineral resources and pollution of ground waters due to nutrient runoff, nutrient recycling fuels sustainable agriculture also on Earth. Exploitation of waste streams for plant production illustrates synergies between space exploration and terrestrial food production, and knowledge on plant responses to different resource utilization scenarios improves the development and design of systems and processes for both Earth and space.

## Data availability statement

The raw data supporting the conclusions of this article will be made available by the authors, without undue reservation.

## Author contributions

ØJ, MS and A-IJ designed the experiment. MS, ØJ, AP and CQ executed the crop tests and performed analyses and calculations. All authors contributed to interpretation of results. MS and ØJ prepared the draft manuscript. All authors contributed to the article and submitted and approved the submitted section.
